# Risks and Benefits of Early Antithrombotic Therapy after Thrombolytic Treatment in Patients with Acute Stroke

**DOI:** 10.1371/journal.pone.0071132

**Published:** 2013-08-08

**Authors:** Sergio Amaro, Laura Llull, Xabier Urra, Víctor Obach, Álvaro Cervera, Ángel Chamorro

**Affiliations:** 1 Functional Unit of Cerebrovascular Diseases, Hospital Clínic, Barcelona, Spain; 2 Institut d’Investigacions Biomediques August Pi i Sunyer, Barcelona, Spain; 3 Medicine Department, School of Medicine, University of Barcelona, Barcelona, Spain; Julius-Maximilians-Universität Würzburg, Germany

## Abstract

**Background:**

Current guidelines recommend withholding antithrombotic therapy (ATT) for at least 24 h in patients with acute ischemic stroke treated with thrombolytic therapy. Herein, we report a retrospective analysis of a single-centre experience on the safety and efficacy of antithrombotic therapy (ATT) started before or after 24 h of intravenous thrombolysis in a cohort of acute ischemic stroke patients.

**Methods:**

A total of 139 patients (Rapid ATT group) received antithrombotic therapy before 24 h of thrombolysis, and 33 patients (Standard ATT group) after 24 h. The brain parenchyma and vessel status were assessed using simple CT scan on admission, multimodal CT scan at the end of thrombolysis, and angio-CT/MRI scan at day 3. Functional outcome was scored using the modified Rankin Scale (mRS) at day 90.

**Results:**

The two ATT groups had similar demographics, stroke subtypes, baseline NIHSS, thrombolytic strategies, vessel-patency rates at the end of thrombolysis, and incidence of bleeding complications at follow up. At day 3, the Rapid ATT group had a non-significant improved vessel-patency rate than the Standard ATT group. At day 90, a greater proportion of patients in the rapid ATT group had shifted down the mRS, and had improved in the NIHSS score.

**Conclusions:**

ATT initiated before 24 h of intravenous thrombolytic therapy in acute stroke patients disclosed no safety concerns compared with a conventional antithrombotic therapy delay of 24 h and showed better functional outcome at follow up. The value of early initiation of ATT after thrombolysis deserves further assessment in randomized controlled trials.

## Introduction

The administration of intravenous recombinant tissue plasminogen activator (rt-PA) is the only specific treatment approved for patients with acute ischemic stroke [1], and current therapeutic guidelines recommend to withhold in these patients the use of anticoagulants or antiplatelet agents as an adjunctive therapy within 24 hours of intravenous thrombolysis [2], [3], in spite of the relatively short plasma half-life (4–6 minutes) of alteplase [4]. However, arterial re-occlusion and formation of fresh thrombi may occur after intravenous thrombolytic therapy [5], and this complication may result in delayed clinical deterioration in some patients [6]. Unfortunately, there is scanty information on the most appropriate timing to administer antithrombotic therapy (ATT) to prevent thrombus progression and arterial re-occlusion after intravenous thrombolysis in acute ischemic stroke, as most Stroke Centers follow protocols derived from the design of the NINDS trial.

Recently, the ARTIS trial showed that the intravenous administration of 300 mg aspirin within 90 minutes after start of intravenous thrombolysis did not improve outcome at 3 months and increased the risk of symptomatic intracerebral hemorrhage (sICH) in patients with acute ischemic stroke [7]. However, the ARTIS trial did not assess the effect of intravenous aspirin on the recanalization rate achieved after thrombolysis. It is also unknown whether the pharmacokinetics of oral antiplatelet agents could ameliorate with respect to intravenous aspirin the safety profile of combined antiplatelet and thrombolytic therapy.

A few phase IV studies suggested that the adjunctive administration of full heparin therapy in stroke patients receiving intravenous thrombolysis did not increase the rate of bleeding complications compared with not anticoagulated historical controls [8], [9]. Unfortunately, these observational studies did not report whether the co-administration of heparin enhanced the reperfusion achieved by rt-PA, reduced the arterial re-occlusion rate at follow up, or improved the long term functional outcome. Therefore, the clinical value of full anticoagulation after intravenous thrombolysis remains to be established.

In light of these unresolved questions, we aimed this study to define the risks and benefits of different time regimens of ATT drugs in ischemic stroke patients treated with intravenous thrombolysis in regular clinical practice at a tertiary Stroke Center.

## Materials and Methods

### Ethics Statement

The study protocol was approved by the Clinical Research Ethics Committee of the Hospital Clínic de Barcelona (CEIC Hospital Clínic). According to local institutional protocols, the patients or their legal representatives signed a written informed consent if age was over 80 years, or treatment was to be initiated >3 h of stroke onset. Because of the retrospective nature of the design, obtaining specific informed consent was not necessary according to local law.

### Study Population

We performed a retrospective analysis from a prospectively collected clinical registry of acute ischemic stroke patients treated with intravenous thrombolytic therapy. The study population included all acute stroke patients younger than 81-years-old treated with intravenous rt-PA at our Comprehensive Stroke Center between October 2008 and June 2011. Overall, 172 patients received rt-PA within 4.5 h of stroke onset, including 33 patients who also received mechanical thrombectomy. During the same study period, 110 patients that received thrombolysis were not included in the study because they were older than 80 (n = 84), experienced an early bleeding complication after intravenous rt-PA therapy and prior to ATT onset (n = 15), or had stroke mimics (n = 11). Recent or current intake of antiplatelet agents (n = 69) was not a limitation to participate in the study as it has been associated with higher rates of early recanalization after thrombolysis [10]. Patients were admitted into an intermediate care Stroke Unit (8 beds) to be managed by certified stroke neurologists following the European Stroke Organization Guidelines [2]. The qualifying strokes were classified according to the Trial of Org 10172 in Acute Stroke Treatment (TOAST) criteria after a complete diagnostic workup [11]. Neurological status was monitored with the National Institutes of Health Stroke Scale (NIHSS) score and the magnitude of neurological recovery was estimated with the formula: [baseline NIHSS−day 90 NIHSS/baseline NIHSS]×100. Functional outcome was quantified with the modified Rankin Scale (mRS) score at 3 months. Demographics, risk factors, laboratory tests, neuroimaging, concomitant therapies, clinical course, and functional outcome were prospectively collected. Data were stored in a local database and declared to a Web-based registry that satisfied all legal requirements for protection of personal data, for monitoring by the Catalan Health Department [12].

### Timing to Initiate the Antithrombotic Therapy

The specific drug and timing to initiate antithrombotic therapy (ATT) after thrombolytic therapy were left at the discretion of the treating physicians of the study. However, a prevailing view was that when heparin was considered indicated its administration should not be delayed more than few hours [13], [14]. Although cardioembolic stroke was a frequent cause to consider the administration of heparin, the anti-inflammatory effects of the drug were other reasons to consider its use in patients with other stroke subtypes [15]. Rapid ATT group included patients in whom ATT was started within 24 h, and Standard ATT, if started beyond 24 h. ATT included oral aspirin (100 mg/day), oral clopidogrel (75 mg/day, preceded by a loading dose of 300 mg in those without prior antithrombotic treatment), or intravenous weight-adjusted unfractionated heparin monitored with the activated partial thromboplastin time (aPTT), as previously reported [16].

### Imaging Protocol

The imaging protocol included a baseline brain CT scan before the onset of thrombolysis, a multimodal CT scan (plain CT/CT perfusion/CT angiography) at the end of rt-PA infusion, a plain CT scan before ATT onset, and a CT/MRI angiography at day 3 of stroke. Before the onset of ATT in patients who received mechanical thrombectomy after systemic thrombolysis, dual energy CT scan was used instead of simple CT scan to differentiate between blood and contrast media extravasation [17]. Additional brain CT scans were performed in patients with an increase on the NIHSS score during hospital admission. All imaging studies were evaluated by investigators blinded to clinical data and timing to ATT.

ASPECT score was assessed on baseline CT as previously described [18]. The bleeding complications were defined on brain imaging according to the European Cooperative Acute Stroke Study (ECASS) criteria as hemorrhagic infarction (HI) and parenchymal hematoma (PH) type 1 and PH type 2 [19]. sICH was defined as any PHs associated with an increment of at least 4 points in the NIHSS score. Vessel patency was graded on CT/MRI angiography according to the Thrombolysis In Myocardial Infarction (TIMI) score [20]. Therefore, patients were grouped into those with vessel-patency (TIMI 2–3) or without (TIMI 0–1), and re-occlusion was defined as a worsening in the score from the end of thrombolysis to day 3 angiography.

### Statistical Analysis

Continuous variables were reported as mean (standard deviation, SD) or median (interquartile range, IQR) and were compared with the Student t test, one-way analysis of variance, Mann–Whitney, or Kruskal-Wallis tests as appropriate. Correlations were assessed with Pearson or Spearman coefficients, and categorical variables were compared with the χ2 and Fisher exact tests. Multivariate logistic regression was adjusted for age, gender, delay to thrombolysis, thrombolytic modality, prior antithrombotic treatment, and variables with a P<0.1 on univariate analysis to assess the effect of rapid versus standard ATT on the bleeding risk and functional outcome. Ordinal regression assessed the overall shift of mRS scores at 3 months. Exploratory analyses were performed to compare the effects of heparin and antiplatelet agents. The analysis was performed using PASW Statistics Version 18.0 and the level of significance was established at a p level of 0.05 (2-sided).

## Results

### Main Characteristics of the Study Population

Overall, 139 (81%) patients were included at a median (IQR) delay of 12 (8–16) hours from thrombolysis in the rapid ATT group, and 33 (19%) patients at a median (IQR) delay of 32 (27–43) hours in the standard ATT group. Both ATT groups had similar demographics, risk factors, stroke subtypes, blood pressure, glucose levels, adjunctive use of mechanical thrombectomy and baseline neurological impairment (Table 1). Correspondingly, the distribution of proximal, distal or tandem arterial occlusions, and the rate of vessel-patency at the end of thrombolytic therapy were similar in both ATT groups (Table 2).

**Table 1:** General characteristics of study population according to ATT onset group.

	Rapid ATT (n = 139)	Standard ATT (n = 33)	p
Age (years), median (IQR)	69 (60–76)	74 (63–78)	0.07
Males, n (%)	85 (61)	20 (61)	0.95
Pre-admission mRS, median (IQR)	0 (0–1)	0 (0–1)	0.82
Diabetes, n (%)	34 (25)	8 (24)	0.98
Smoking, n (%)	41 (30)	7 (21)	0.34
Hypertension, n (%)	92 (66)	25 (76)	0.29
Dyslipidemia, n (%)	60 (43)	12 (36)	0.48
Atrial Fibrillation, n (%)	29 (21)	9 (27)	0.43
Ischemic Heart Disease, n (%)	21 (15)	4 (12)	0.66
Peripheral Vascular Disease, n (%)	8 (6)	2 (6)	0.95
Previous stroke, n (%)	14 (10)	5 (15)	0.40
Previous antithrombotic use, n (%)	53 (38)	16 (49)	0.27
TOAST			0.81
Cardioembolism, n (%)	50 (36)	11 (33)	
Aterothrombotic, n (%)	28 (20)	5 (15)	
Lacunar, n (%)	16 (12)	4 (12)	
Undetermined, n (%)	33 (24)	11 (33)	
Other etiologies, n (%)	12 (8)	2 (6)	
Baseline Systolic BP (mmHg), median (IQR)	155 (140–170)	160 (131–170)	0.80
Baseline glucose (mg/dl), median (IQR)	124 (109–154)	119 (106–140)	0.54
Systemic rtPA plus endovascular tx, n (%)	28 (20)	5 (15)	0.51
Time to rtPA treatment (min), median (IQR)	123 (95–185)	130 (100–180)	0.80
ASPECT score at baseline CT, median (IQR)	10 (8–10)	10 (9–10)	0.40
Baseline NIHSS, median (IQR)	6 (3–14)	8 (4–12)	0.54
NIHSS at 24 h, median (IQR)	3 (1–8)	4 (0–15)	0.64
NIHSS at day 7, median (IQR)	2 (0–6)	3 (0–13)	0.24

**Table 2:** Vessel status at baseline and at 72 h in both att groups.

	RapidATT	StandardATT	p
**Vessel at end of Thrombolysis**	N = 136	N = 33	**0,60**
Patent vessel, n (%)	74 (54)	20 (61)	
Proximal occlusion, n (%)	32 (24)	8 (24)	
Distal occlusion, n (%)	15 (11)	4 (12)	
Tandem occlusion, n (%)	15 (11)	1 (3)	
**Vessel patency at day 3**	N = 132	N = 28	**0,06**
TIMI 2–3, n (%)	126 (95)	25 (89)	
TIMI 0–1, n (%)	6 (5)	3 (11)	
**Vessel re-occlusion at day 3**	N = 71	N = 18	**0,20**
No, n (%)	71 (100)	17 (94)	
Yes, n (%)	0(0)	1 (6)	

Heparin or antiplatelet agents (aspirin/clopidogrel) were selected in 87 (63%) and 52 (37%) patients in the rapid ATT group, and in 8 (24%) and 25 (76%) patients in the standard ATT group, p<0.001. Overall, heparin was initiated more rapidly than the antiplatelet agents in the study [median (IQR) 11.7 h (8.1–15.8) versus 18.4 (11.6–26.8), p<0.001].

### Vessel Status at follow up in Relation to the Delay of ATT

As shown in table 2, the vessel-patency rate (TIMI 2–3) at day 3 was non-significantly increased in the rapid ATT group compared with the standard ATT group (p = 0.06). In exploratory analyses of patients with vessel-patency at the end of thrombolysis (n = 94), only one patient in the standard ATT group experienced vessel re-occlusion (Table 2).

### Delay to ATT and Clinical Outcome

The crude recovery of NIHSS score at day 90 was inversely related with the delay to ATT (β = −.248, p = 0.001), and the association remained after adjustment for age, gender, and baseline NIHSS score (β = −.236, p = 0.002). Overall, the NIHSS score recovery at day 90 was 89% (63%–100%) in the rapid ATT group and 65% (12%–100%) in the standard ATT group (p = 0.04).

**Figure 1:**
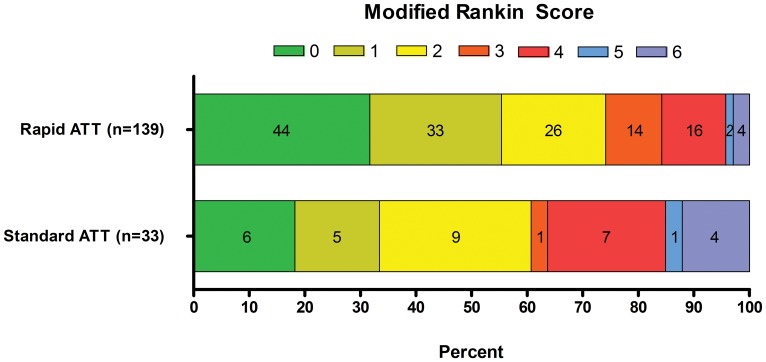
Modified Rankin Scale score distribution at day 90 of stroke according to the delay to initiate antithrombotic therapy.

**Figure 2:**
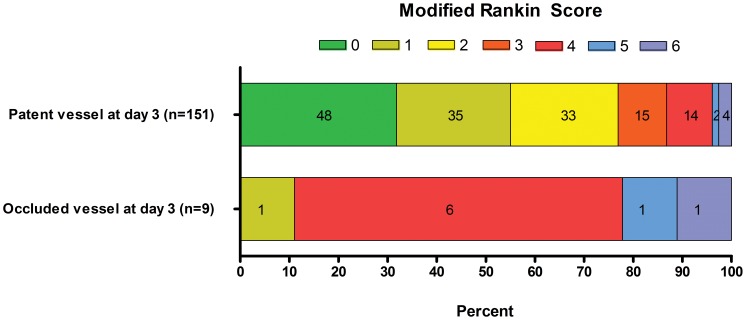
Modified Rankin Scale score distribution at day 90 of stroke according to presence of patent vessel (TIMI 2–3) or occluded vessel (TIMI 0–1) at day 3.

The median (IQR) mRS score at day 90 was lower in the rapid ATT group than in the standard ATT group, 1 (0–3) and 2 (1–4), p = 0.01. More patients in the rapid ATT group shifted down in the mRS at day 90, p = 0.01 (Figure 1), and the effect was significant in ordinal regression analysis (Table 3). Expectedly, the mRS score at day 90 was strongly associated with vessel status at day 3, p<0.001 (Figure 2).

**Table 3:** Multivariate analysis of outcome.

	Binary LR OR (CI 95%); p	Ordinal LR OR (CI 95%); p
**Age (per 10 years)**	1.08 (0.76–1.53); p = 0.69	0.99 (0.76–1.29); p = 0.92
**Baseline NIHSS (IQR)**	0.36 (0.23–0.58); p<0.001	0.40 (0.28–0.57); p<0.001
**ASPECT score at baseline CT (per unit)**	1.07 (0.70–1.64); p = 0.76	1.27 (0.96–1.70); p = 0.09
**Sex (male)**	1.08 (0.48–2.45); p = 0.86	1.22 (0.66–2.25); p = 0.52
**Prior mRS (per unit)**	0.66 (0.42–1.02); p = 0.06	0.66 (0.48–0.91); p = 0.01
**TOAST (per category)**	1.58 (1.19–2.10); p = 0.002	1.41 (1.11–1.75); p = 0.002
**Glucose (IQR)**	0.70 (0.50–0.99); p = 0.04	0.72 (0.55–0.93); p = 0.01
**Dislypidemia (yes vs no)**	0.41 (0.17–1.99); p = 0.05	0.70 (0.37–1.33); p = 0.28
**Trombolytic modality (MT plus IVT, vs IVT alone)**	2.85 (0.86–9.45); p = 0.09	2.08 (0.89–4.84); p = 0.09
**Prior antihrombotic treatment (yes vs no)**	2.12 (0.89–5.07); p = 0.09	1.34 (0.71–2.52); p = 0.37
**Rapid ATT (vs. standard ATT)**	4.35 (1.57–12.08); p = 0.005	3.43 (1.65–7.13); p = 0.001

IVT = Intravenous thrombolysis; LR = Logistic regression; mRS = Modified Rankin Scale; MT = Mechanical thrombectomy; NIHSS = National Institutes of Health Stroke Scale; TOAST = Trial of Org 10172 in Acute Stroke Treatment.

Binary LR: OR for excellent outcome (mRS 0–1 vs 2–6); Ordinal LR: OR for shifting down on the mRS score.

### Delay to ATT and Adverse Events

Hemorrhagic complications were detected in 17 (10%) patients at follow up, including 9 (5%) HI, and 8 (5%) PH type I or II, although only 3 (2%) patients had a sICH. There were no significant differences in hemorrhagic risk between the ATT groups (Table 4), or between patients treated with intravenous weight adjusted heparin or oral antiplatelet agents (Table S1). In patients with recent intake of antiplatelet agents at the time of the qualifying stroke (n = 69) there were no significant differences in clinical findings, vessel-patency rates at end of thrombolysis, vessel-patency at day 3, or incidence of bleeding complications compared with naïve patients (Table S2).

**Table 4:** Bleeding complications on brain imaging at day 3.

	RapidATT	StandardATT	p
**Study cohort**	N = 139	N = 33	
Any Hemorrhagic Transformation, n (%)	16 (12)	1 (3)	0.20
Hemorrhagic infarction, n (%)	9 (6)	0 (0)	0.21
Parenchymal hematoma, n (%)	7 (5)	1 (3)	1.00
Symptomatic ICH after ATT onset, n (%)	3 (2)	0 (0)	1.00
**Heparin cohort**	N = 87	N = 8	
Any Hemorrhagic Transformation, n (%)	10 (11)	0 (0)	0.59
Hemorrhagic infarction, n (%)	5 (6)	0 (0)	1.00
Parenchymal hematoma, n (%)	5 (6)	0 (0)	1.00
Symptomatic ICH after ATT onset, n (%)	2 (2)	0 (0)	1.00
**Antiplatelets**	N = 52	N = 25	
Any Hemorrhagic Transformation, n (%)	6 (12)	1 (3)	0.42
Hemorrhagic infarction, n (%)	4 (8)	0 (0)	0.30
Parenchymal hematoma, n (%)	2 (4)	1 (4)	1.00
Symptomatic ICH after ATT onset, n (%)	1 (2)	0 (0)	1.00

## Discussion

This retrospective study analyzed the risks and benefits associated with the delay to initiate ATT in prospectively registered patients with acute ischemic stroke treated with intravenous thrombolytic therapy. To our knowledge, this is the first observational study providing a detailed description of vessel status at follow up, unlike previous phase IV studies [8], [9], or randomized phase III clinical trials [7]. By means of repeated imaging acquisition, the study showed a trend towards greater vessel-patency rate at day 3 in patients that received ATT before 24 h of thrombolysis, despite that the anatomical location of the occluded vessels at onset was comparable with the occlusions identified in patients that received ATT beyond 24 h. The overall rate of vessel-patency at the end of thrombolysis was 55% and did not show significant differences between the ATT groups. This vessel-patency rate was slightly higher than the 43% observed in 14 pooled intravenous thrombolysis studies [21], and most likely responded to the fact that almost 20% of the patients received mechanical thrombectomy once the monitorization of the vessel status with CT angiography showed that systemic thrombolysis had failed to achieve a full recanalization. Reassuringly, the two ATT groups had a similar proportion of patients treated with mechanical thrombectomy.

The overall vessel-patency rate at day 3 of thrombolysis increased to 88% in the study, and the overall re-occlusion rate observed in the current study was very low and notably absent in the rapid ATT group. These findings were indicative that many patients experienced delayed recanalization, and very few arterial reocclusion. For vessel-patency at day 3 was associated with an improved functional outcome at 3 months it is likely that most recanalizations occurred shortly after the end of thrombolysis. Interestingly, the rapid ATT group showed a trend towards increased vessel-patency compared with the conventional ATT group, suggesting that a more rapid initiation of ATT prevented delayed thrombus progression. In the current study, the very low re-occlusion rate observed in standard (6%) and rapid ATT (0%) groups opposed previous continuous transcranial Doppler monitoring studies in which re-occlusion occurred in 20–34% of rt-PA treated patients [6]. The low re-occlusion rate of the study might reflect the addition of rescue mechanical thrombectomy in patients resistant to systemic thrombolysis.

Overall, the safety profile of early ATT onset observed in our study is congruent with several phase IV studies suggesting that the adjunctive administration of full heparin therapy in stroke patients receiving intravenous thrombolysis did not increase the rate of bleeding complications compared with not anticoagulated historical controls [8], [9]. More recently, the ARTIS trial showed that the early administration of 300 mg aspirin after systemic alteplase use in acute ischemic stroke patients increased the risk of sICH [7], consistently with results from a previous trial that assessed the combination of aspirin with streptokinase [22]. In the ARTIS trial, aspirin was administered intravenously and within 90 minutes after start of alteplase treatment. In our study, early ATT was started within a median (IQR) of 12 (8–16) hours from thrombolytic treatment, which is far away from the plasmatic half-life of alteplase [4]. Moreover, prior to ATT onset a plain CT was mandatory to confirm the absence of early bleeding complications related to thrombolytic treatment. Finally, in our study, baseline NIHSS score in rapid ATT group (median (IQR) 6 (3–14)) was lower compared with other studies such as the ARTIS trial (median (IQR) 9 (5–15)). Whether initial stroke severity could influence the safety and efficacy profile of early ATT onset after alteplase treatment is unknown and deserves further study in controlled trials.

Reassuringly, the safety of the study was assessed by monitors from the Health Department and confirmed that the rapid ATT group did not manifest an increased risk of symptomatic or asymptomatic bleedings. Furthermore, the rapid ATT group disclosed at day 90 a better functional outcome in the mRS score adjusted for confounders, as well as greater neurological recovery in the NIHSS score. According to the retrospective nature of the study, the lack of randomization of the time to start ATT was a limitation of the study although the ATT groups did not have significant differences at baseline. In addition this limitation was further addressed by including in the analysis other potential confounders which have shown to predict poor 3-month long-term outcome in ischemic stroke patients treated with rt-PA [23]-[25]. Therefore, our findings might suggest that the early implementation of ATT was neuroprotective.

From a clinical perspective our results indicated that up to 80% of thrombolysed patients received the first dose of ATT before 24 h of thrombolysis regardless of current recommendations. Nonetheless, it was also noticeable that the treating physicians diverged in their interpretation of the ideal patient that should follow the guidelines, as no apparent demographic trait, clinical finding or angiographic characteristic identified the patient suitable for each ATT group, except for a trend for older age in patients allocated in the standard ATT group. However, the association between rapid ATT and better prognosis remained significant in multivariate models adjusted by the effect of age. On exploratory analyses, we did not identify significant differences in efficacy or safety between heparin and oral antiplatelet agents but we cannot exclude a type II error. Indeed, a majority of patients received full intravenous anticoagulation in the rapid ATT group, while oral antiplatelets prevailed in the conventional ATT group, and this misbalance could have potentially influenced the observed trend towards an increased vessel patency rate in patients included in the rapid ATT group. This unbalanced treatment allocation accorded with previous studies which emphasized the importance of accelerating the initiation of heparin therapy in suitable patients [13], [14], to optimize the anti-inflammatory effects of the drug [26], and reduce the risk of early stroke recurrence [16].

### Conclusions

Collectively, this study challenged the current recommendation of withholding ATT for at least 24 h in patients with acute ischemic stroke treated with thrombolytic therapy. Alternatively, it suggested that the overall risk benefit of antithrombotic drugs was improved with a swifter treatment onset. The value of early initiation of ATT in thrombolysed patients should be confirmed in randomized controlled studies.

## Supporting Information

Table S1 **General characteristics of study population according to the antithrombotic drug used.**
(DOC)

Table S2 **General characteristics of study population according to the pre-stroke antithrombotic treatment.**
(DOC)
