# Supplementary material for: Risks and Benefits of Early Antithrombotic Therapy after Thrombolytic Treatment in Patients with Acute Stroke
Source: PLoS One. 2013 Aug 8;8(8):e71132. doi: 10.1371/journal.pone.0071132 (PMC3738638; doi:10.1371/journal.pone.0071132)
Supplement: Table S1 — General characteristics of study population according to the antithrombotic drug used. (DOC) [file pone.0071132.s001.doc]

**Table S**1: General characteristics of study population according to the antithrombotic drug used.

|  | **Heparin**  **(n=95)** | **Antiplatelets**  **(n=77)** | **p** |
| --- | --- | --- | --- |
| Age (years), median (IQR) | 71 (65-76) | 69 (58-76) | 0.09 |
| Males, n (%) | 56 (59) | 49 (64) | 0.53 |
| Pre-admission mRS, median (IQR) | 0 (0-1) | 0 (0-1) | 0.13 |
| Diabetes, n (%) | 21 (22) | 21 (27) | 0.43 |
| Smoking, n (%) | 25 (26) | 23 (30) | 0.61 |
| Hypertension, n (%) | 64 (67) | 53 (69) | 0.84 |
| Dyslipidemia, n (%) | 41 (43) | 31 (40) | 0.70 |
| Atrial Fibrillation, n (%) | 31 (33) | 7 (9) | <0.001 |
| Ischemic Heart Disease, n (%) | 16 (17) | 9 (12) | 0.34 |
| Peripheral Vascular Disease, n (%) | 5 (5) | 5 (7) | 0.73 |
| Previous stroke, n (%) | 10 (11) | 9 (12) | 0.81 |
| Previous antithrombotic use, n (%) | 41 (43) | 28 (36) | 0.37 |
| TOAST |  |  | <0.001 |
| Cardioembolism, n (%) | 52 (55) | 9 (12) |  |
| Aterothrombotic, n (%) | 19 (20) | 14 (18) |  |
| Lacunar, n (%) | 0 (0) | 20 (26) |  |
| Undetermined, n (%) | 19 (20) | 25 (33) |  |
| Other etiologies, n (%) | 5 (5) | 9 (12) |  |
| Baseline Systolic BP (mmHg), median (IQR) | 150 (135-170) | 163 (143-173) | 0.08 |
| Baseline glucose (mg/dl), median (IQR) | 121 (106-162) | 122 (104-143) | 0.65 |
| Systemic rtPA only, n (%) | 74 (78) | 65 (84) | 0.28 |
| Systemic rtPA plus endovascular treatment, n (%) | 21 (22) | 12 (16) | 0.28 |
| Time to rtPA treatment (min), median (IQR) | 125 (96-182) | 124 (95-185) | 0.80 |
| Time to ATT onset, median (IQR) | 12 (9-16) | 18 (12-27) | <0.001 |
| ASPECT score at baseline CT, median (IQR) | 9 (8-10) | 10 (9-10) | 0.09 |
| Baseline NIHSS, median (IQR) | 8 (4-14) | 6 (3-12) | 0.34 |
| NIHSS at 24h, median (IQR) | 4 (1-9) | 3 (0-7) | 0.15 |
| NIHSS at day 7, median (IQR) | 2 (0-6) | 2 (0-4) | 0.27 |
| NIHSS at day 90, median (IQR) | 1 (0-4) | 0 (0-3) | 0.29 |
| mRS at day 90, median (IQR) | 1 (0-4) | 2 (0-3) | 0.27 |
| mRS 0-1 day 90, n (%) | 45 (47) | 43 (56) | 0.27 |
| Symptomatic ICH after ATT onset, n (%) | 2 (2) | 1 (1) | 1.00 |
| Vessel status at end of Thrombolysis |  |  | 0.004 |
| Patent vessel, n/n assessed (%) | 43/93 (46) | 51/76 (67) |  |
| Proximal occlusion, n/n assessed (%) | 25/93 (27) | 15/76 (20) |  |
| Distal occlusion, n/n assessed (%) | 17/93 (18) | 2/76 (3) |  |
| Tandem occlusion, n/n assessed (%) | 8/93 (9) | 8/76 (11) |  |
| Vessel patency at day 3 |  |  | 0.59 |
| TIMI 2-3, n/n assessed (%) | 84/89 (94) | 67/71 (94) |  |
| TIMI 0-1, n/n assessed (%) | 5/89 (6) | 4/71 (6) |  |
| Vessel re-occlusion at day 3 |  |  | 0.46 |
| Yes, n/n assessed (%) | 1/41 (2) | 0/48 (0) |  |
