# Supplementary material for: Risks and Benefits of Early Antithrombotic Therapy after Thrombolytic Treatment in Patients with Acute Stroke
Source: PLoS One. 2013 Aug 8;8(8):e71132. doi: 10.1371/journal.pone.0071132 (PMC3738638; doi:10.1371/journal.pone.0071132)
Supplement: Table S2 — General characteristics of study population according to the pre-stroke antithrombotic treatment. (DOC) [file pone.0071132.s002.doc]

**Table S2: General characteristics of study population according to the pre-stroke antithrombotic treatment.**

| **Prior antithrombotic treatment** | **yes**  **(n=69)** | **no**  **(n=103)** | **p** |
| --- | --- | --- | --- |
| Age (years), median (IQR) | 73 (67-77) | 68 (58-74) | 0.006 |
| Males, n (%) | 46 (67) | 59 (57) | 0.22 |
| Pre-admission mRS, median (IQR) | 0 (0-1) | 0 (0-1) | 0.02 |
| Diabetes, n (%) | 24 (35) | 18 (18) | 0.01 |
| Smoking, n (%) | 20 (29) | 28 (27) | 0.80 |
| Hypertension, n (%) | 60 (87) | 57 (55) | <0.001 |
| Dyslipidemia, n (%) | 41 (59) | 31 (30) | <0.001 |
| Atrial Fibrillation, n (%) | 22 (32) | 16 (16) | 0.02 |
| Ischemic Heart Disease, n (%) | 21 (30) | 4 (4) | <0.001 |
| Peripheral Vascular Disease, n (%) | 8 (12) | 2 (2) | 0.02 |
| Previous stroke, n (%) | 18 (26) | 1 (1) | <0.001 |
| Antithrombotic drug used |  |  |  |
| Antiplatelets, n (%) | 61 (88) | - | - |
| Anticoagulants, (%) | 8 (12) | - | - |
| TOAST |  |  | 0.06 |
| Cardioembolism, n (%) | 28 (41) | 33 (32) |  |
| Aterothrombotic, n (%) | 16 (23) | 17 (17) |  |
| Lacunar, n (%) | 4 (6) | 16 (16) |  |
| Undetermined, n (%) | 19 (28) | 25 (24) |  |
| Other etiologies, n (%) | 2 (3) | 12 (12) |  |
| Baseline Systolic BP (mmHg), median (IQR) | 150 (134-168) | 156 (140-175) | 0.05 |
| Baseline glucose (mg/dl), median (IQR) | 126 (110-163) | 118 (104-146) | 0.16 |
| Systemic rtPA only, n (%) | 54 (78) | 85 (83) | 0.49 |
| Systemic rtPA plus endovascular tx, n (%) | 15 (22) | 18 (18) | 0.49 |
| Time to rtPA treatment (min), median (IQR) | 125 (100-180) | 123 (93-185) | 0.53 |
| Time to ATT onset, median (IQR) | 16 (9-23) | 15 (9-19) | 0.46 |
| ASPECT score at baseline CT, median (IQR) | 10 (9-10) | 10 (8-10) | 0.58 |
| Baseline NIHSS, median (IQR) | 6 (4-12) | 7 (4-15) | 0.53 |
| NIHSS at 24h, median (IQR) | 3 (1-7) | 3 (1-7) | 0.55 |
| NIHSS at day 7, median (IQR) | 2 (0-6) | 2 (0-7) | 0.53 |
| NIHSS at day 90, median (IQR) | 1 (0-3) | 1 (0-4) | 0.71 |
| mRS at day 90, median (IQR) | 1 (0-3) | 1 (0-3) | 0.92 |
| mRS 0-1 day 90, n (%) | 36 (52) | 52 (51) | 0.83 |
| Symptomatic ICH after ATT onset, n (%) | 0 (0) | 3 (3) | 0.28 |
| Bleeding complications on brain imaging at day 3 |  |  |  |
| Hemorrhagic infarction, n (%) | 3 (4) | 6 (6) | 0.74 |
| Parenchymal hematoma, n (%) | 2 (3) | 6 (6) | 0.47 |
| Any Hemorrhagic Transformation, n (%) | 5 (7) | 12 (12) | 0.34 |
| Vessel status at end of Thrombolysis |  |  | 0.62 |
| Patent vessel, n/n assessed (%) | 37/68 (54) | 57/101 (56) |  |
| Proximal occlusion, n/n assessed (%) | 14/68 (21) | 26/101 (26) |  |
| Distal occlusion, n/n assessed (%) | 10/68 (15) | 9/101 (9) |  |
| Tandem occlusion, n/n assessed (%) | 7/68 (10) | 9/101 (9) |  |
| Vessel patency at day 3 |  |  | 0.69 |
| TIMI 2-3, n/n assessed (%) | 62/65 (95) | 89/95 (94) |  |
| TIMI 0-1, n/n assessed (%) | 3/65 (5) | 6/95 (6) |  |
| Vessel re-occlusion at day 3 |  |  | 1.00 |
| Yes, n/n assessed (%) | 0/35 (0) | 1/54 (2) |  |
